# 分子印迹固相微萃取结合色谱/质谱在药物残留测定中的应用

**DOI:** 10.3724/SP.J.1123.2025.06034

**Published:** 2026-02-08

**Authors:** Jingyi YAN, Jingying HUANG, Siyuan PENG, Mingsan MAN, Dani SUN, Ping LIU, Lingxin CHEN, Jinhua LI, Huaying FAN

**Affiliations:** 1. 烟台大学药学院，新型制剂与生物技术药物研究山东省高校协同创新中心，分子药理和药物评价教育部 重点实验室，山东 烟台 264005; 1. School of Pharmacy，Yantai University，Collaborative Innovation Center of Advanced Drug Delivery System and Biotech Drugs in Universities of Shandong，Key Laboratory of Molecular Pharmacology and Drug Evaluation，Ministry of Education，Yantai 264005，China; 2. 中国科学院烟台海岸带研究所，山东省海岸带环境过程重点实验室，海岸带生态环境监测技术与装备山东省工程研究中心，山东 烟台 264003; 2. Coastal Zone Ecological Environment Monitoring Technology and Equipment Shandong Engineering Research Center，Shandong Key Laboratory of Coastal Environmental Processes，Yantai Institute of Coastal Zone Research，Chinese Academy of Sciences，Yantai 264003，China; 3. 绍兴文理学院高等研究院，浙江 绍兴 312000; 3. Institute for Advanced Study，Shaoxing University，Shaoxing 312000，China

**Keywords:** 药物残留, 固相微萃取, 分子印迹, 样品前处理, 制备技术, 应用, drug residues, solid-phase microextraction, molecular imprinting, sample pretreatment, preparation techniques, applications

## Abstract

药物广泛应用于医疗、农业及畜牧业领域，然而长期和不规范使用致使其在食品、生物及环境中残留，对人类健康和生态环境构成潜在严重威胁。发展灵敏、精准的药物残留检测方法已成为药物研究的重要前提和当前热点。然而，待测样品基质复杂且药物残留水平极低，因此即便是色谱/质谱这类高效技术，也仍需依赖高效的样品前处理环节。分子印迹固相微萃取（MI-SPME）兼顾固相微萃取快速、高效、无溶剂的优点，以及分子印迹聚合物（MIPs）的特异性识别与选择性吸附能力，在复杂样品药物残留的高选择性分离和萃取方面展现出显著优势。本综述聚焦2019年以来MI-SPME结合色谱/质谱用于药物残留测定的研究进展。首先，介绍了SPME的原理与操作流程，以及用于SPME的MIPs的制备方法和新技术/策略，包括自由基聚合、原位聚合、溶胶-凝胶聚合、表面印迹、纳米印迹、虚拟模板印迹、多模板印迹、多功能单体印迹和刺激响应型印迹。然后，概述了6种不同的MI-SPME装置模式，即基于MIPs的涂层纤维式、管内式、搅拌棒式、分散式、薄膜式和尖端式SPME。随后，重点总结了MI-SPME结合色谱/质谱在食品安全、环境监测、生物医药领域的典型应用。最后，讨论了MI-SPME在药物残留测定中面临的挑战，如MIPs的制备与优化、MI-SPME模式创新、新材料开发与成本控制、自动化集成等，并对MI-SPME制备和药物残留检测应用的前景进行了展望。

## Abstract (en)

Drugs play an indispensable role in the fields of medicine， agriculture， and animal husbandry. However， their long-term and improper use may lead to drug residues in food， the environment and organisms， posing a potentially serious threat to human health and the ecological environment. For instance， antibiotic residues may induce bacterial resistance， pesticide residues may cause neurotoxicity， and hormone drugs may interfere with the endocrine system. Therefore， developing sensitive and accurate detection methods for drug residues has become an important prerequisite and current hot topic in drug research. Meanwhile， the complicated matrices and low contents of the residues make it necessary for the widely used chromatography/mass spectrometry （MS） determination technologies to be coupled with efficient sample pretreatment procedures. Molecularly imprinted solid-phase microextraction （MI-SPME） technology combines the rapidity， high efficiency and solvent-free characteristics of SPME， and the specific recognition and selective adsorption capabilities of molecularly imprinted polymers （MIPs）， and shows significant advantages in the highly selective separation and enrichment of drug residues in complex samples. In recent years， the MI-SPME technology has become a research hotspot in the field of drug residue detection.This work systematically reviews the research progress since 2019 on the application of MI-SPME coupled with chromatography/MS in drug residue detection across food safety， environmental monitoring and biomedical fields. First， this work introduces in detail on the working principle and operation process of SPME technology. SPME achieves efficient enrichment of target analytes through the selective adsorption of the stationary phase-coated fibers， offering simplicity， speed， minimal solvent use， and compatibility with analytical instruments such as chromatography/MS.Next， the review focuses on elaborating the preparation methods and new technologies and strategies of MIPs. The traditional methods for preparing MIPs mainly include free radical polymerization， in-situ polymerization and sol-gel methods. However， traditional MIPs have defects such as template leakage risk， limited binding ability， and irregular material morphology， which restrict the application range. To this end， researchers have developed a series of novel preparation technologies and strategies， such as surface imprinting， nanoimprinting， dummy template imprinting， multi-template imprinting， multifunctional monomer imprinting and stimulus-response imprinting. These technologies and strategies have significantly enhanced the recognition and enrichment ability of MIPs for trace drug residues in complex samples by optimizing their structures and performances.To meet the requirements of different sample types and analytical instruments， MI-SPME media need to be designed into specific technical configurations through chemical or physical methods. This review summarizes six different MI-SPME device modes： MIPs-coated fiber SPME， MIPs in-tube SPME （IT-SPME）， MIPs stir bar sorptive extraction （SBSE）， MIPs dispersive SPME （DSPME）， MIPs thin-film SPME （TFME）， and MIPs in-tip SPME. Each mode offers unique advantages for the separation， enrichment and determination of drug residues in real samples. For example， the coated fiber SPME is simple to operate and suitable for direct immersion or headspace extraction of liquid samples； IT-SPME features miniaturization and automation， with excellent compatibility with chromatographic and mass spectrometric systems； DSPME achieves efficient separation and enrichment by dispersing adsorbents directly into sample solutions.Then， the applications of MI-SPME in the fields of food safety， environmental monitoring and biological medicine are summarized， highlighting typical research examples. In the field of food safety， MI-SPME can be used to detect pesticide residues， veterinary drug residues， and drugs for human use in fruits， vegetables， animal meats and dairy products. In environmental monitoring， it can be used for the detection of drug residues in aqueous environments and soil. In the field of biological medicine， it can be used for the analysis of drug residues in biological samples such as plasma， urine， and serum.Although the MI-SPME technology has shown great potential in drug residue detection， it still faces some challenges. For example， the preparation process of MIPs needs to be further optimized to improve their selectivity and stability； the development and application of new materials （such as graphene， metal-organic frameworks） for composite MIPs still need to solve problems such as high cost and complex processes； the integration of MI-SPME technology and automated equipment is also a bottleneck and important direction for future development. Looking ahead， with the advancement of green chemistry principles and point-of-care testing technologies， MI-SPME is expected to play an even greater role in drug residue detection. It will provide more efficient and precise technical support for food safety， environmental monitoring， and biomedical research.

药物在畜牧业、农业及现代医疗领域发挥着关键作用，例如，在农业和畜牧业中，合理使用农药和兽药能有效控制病虫害，预防动物疾病，从而保障农作物产量和畜禽的健康生长^［1］^。在人类医疗领域，药物是治疗疾病、缓解疼痛、预防感染的重要手段。然而，药物的长期广泛应用及不规范使用，导致其在食品、生物及环境介质中残留累积，对生态系统和人体健康造成严重威胁^［2-4］^。例如，在兽药残留方面，抗生素类如四环素（TCs）不仅会诱导细菌耐药性，还可能引发超敏反应，降低临床疗效^［5］^；激素类如17*β*-雌二醇（E2）会干扰内分泌系统，影响生长发育与生殖功能；抗寄生虫药如苯并咪唑类，则可能产生神经毒性，引发头晕等症状。农药残留中，有机磷类杀虫剂（OPPs）如乙硫磷通过抑制胆碱酯酶导致神经毒性，严重损害中枢神经系统；除草剂如2，4-二氯苯氧乙酸（2，4-D）会干扰动植物生理代谢，破坏土壤生态平衡；杀菌剂如吡丙醚可能影响非靶标生物的繁殖发育。人用药物，如羟考酮、普萘洛尔，前者可能增加人体药物成瘾风险、影响神经系统，后者干扰心血管系统调节、影响心脏功能^［6，7］^。这些药物均在食品、生物及环境介质中被检出。因此，建立有效的药物残留监测体系至关重要。

在多种多样的检测技术中，色谱/质谱技术展现出独特优势，已成为复杂基质中药物残留测定的主要手段。色谱技术，包括高效液相色谱-紫外（HPLC-UV）、高效液相色谱-二极管阵列检测器（HPLC-DAD）、超高效液相色谱-光电二极管阵列检测器（UHPLC-PDA）、气相色谱-电子捕获检测器（GC-ECD）、气相色谱-氮磷检测器（GC-NPD）、气相色谱-氢火焰离子化检测器（GC-FID）等，其核心在于利用物质在固定相和流动相之间分配系数的差异实现复杂混合物中各组分的有效分离，能有效克服基质背景干扰。质谱技术（MS），如电喷雾电离质谱（ESI-MS）以及离子迁移谱（IMS）等，则通过将样品分子离子化，按质荷比（*m/z*）进行分离和检测，提供化合物精确的相对分子质量及结构碎片信息，为待测物的结构确证和高特异性定量提供了强有力的工具。将色谱强大的分离能力与质谱卓越的定性和定量能力相结合，形成的色谱-质谱联用技术，如液相色谱-串联质谱（LC-MS/MS）、超高效液相色谱-串联质谱（UHPLC-MS/MS）、GC-MS、GC-MS/MS，能够对复杂样品中痕量乃至超痕量的目标药物残留物进行高灵敏、高选择且可靠的定性和定量分析^［8-11］^。

尽管如此，色谱/质谱检测技术仍然需要结合样品前处理。药物残留的浓度通常较低，而且复杂基质和干扰成分会对药物残留的分离和定量分析产生严重影响，并损害仪器^［12］^。因此，样品前处理作为分析过程的关键环节，能够将目标物从复杂的基质中分离并富集，从而提高分析检测的灵敏度和准确性^［13-15］^。固相微萃取（SPME）是一种基于样品基质-固定相分配平衡原理的样品前处理技术^［16］^，通过固定相涂层纤维的选择性吸附实现对目标物的高效富集^［17］^。SPME集采样、萃取、预富集、进样于一体，操作简便，耗时短，几乎无需使用有机溶剂^［18，19］^，方便与HPLC等各种分析仪器配对且更易实现自动化^［20］^，在药物残留检测领域具有广阔的应用前景。固体吸附剂是影响SPME效率的关键因素，但目前商品化的吸附剂种类有限，缺乏选择性，传统吸附剂通常无法有效提取复杂基质中的目标药物残留。因此，亟需开发高选择性的吸附剂^［21］^。

近年来，通过分子印迹技术（MIT）制备的分子印迹聚合物（MIPs）越来越多地用于复杂基质中目标物的选择性识别和吸附^［22］^。MIPs是基于模板分子可控合成的高分子材料，通过“锁-钥”机制形成与模板分子在空间结构（形状、尺寸）与化学功能基团上高度匹配的特异性识别位点，从而赋予其对模板目标物的高选择性。同时，MIPs兼具优异的物理化学稳定性和制备简单、成本低等特点，使其在复杂基质低含量目标物的精准捕获方面应用广泛。将MIPs作为SPME的吸附剂构建的分子印迹固相微萃取（MI-SPME）技术，既保留了MIPs的选择性识别能力和稳健性，又融合了SPME技术的快速、高效、操作便捷、溶剂消耗少及绿色环保等优势，能够进行高效识别和富集^［23］^。因此，MI-SPME已广泛用于复杂样品中药物残留的选择性分离和富集^［24-26］^。例如，Rahimi等^［27］^开发了一种新型的MI-SPME纤维，结合HPLC-UV，用于尿液样本中苯巴比妥的测定。Xiang等^［28］^制备了一种新型MI-SPME涂层纤维，结合GC-NPD，应用于果蔬中OPPs的分析。

尽管MIPs结合SPME技术已在药物残留测定中取得较多研究成果，但目前针对MI-SPME应用于药物残留检测领域的综述较少。Sarafraz-Yazdi等^［26］^在2015年对MIPs在SPME技术的应用进行了梳理。薛伟亮等^［29］^在2020年综述了MI-SPME用于食品安全中痕量农药和兽药残留测定的研究进展。Martins等^［30］^在2024年总结了MI-SPME在生物医药样品中药物化合物分析的应用。鉴于此，本文综述了2019年以来MI-SPME结合色谱/质谱应用于食品安全、环境监测、生物医药领域中药物残留测定的研究新进展，主要内容如图1所示。首先介绍了SPME的原理与操作流程、MIPs的制备方法与新技术/策略以及不同MI-SPME模式的特点，然后重点总结了MI-SPME提取食品、环境和生物样品等复杂基质中药物残留的代表性应用，最后提出了MI-SPME在药物残留测定中可能面临的挑战并展望了MI-SPME的发展和应用。

**图1:**
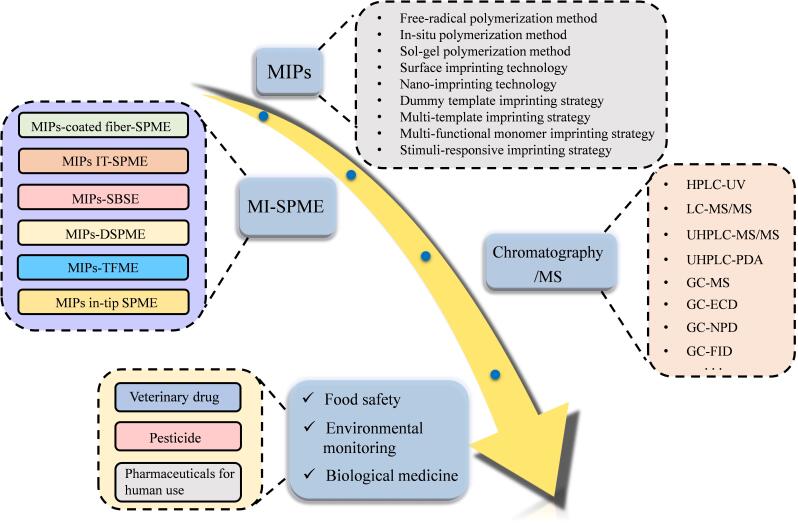
本文主要内容示意图

## 1 MI-SPME的基本元素

MI-SPME技术的核心在于SPME操作模式与MIPs制备的协同优化。这一过程需要根据样品基质特性、目标物性质和分析需求，选择合适的SPME模式，并针对目标分子结构设计具有特定识别性能的MIPs材料。二者的合理结合是实现高效选择性萃取的关键。

### 1.1 SPME技术

在SPME过程中，关键环节是制备SPME纤维，它由一层极薄的熔融石英纤维构成，表面涂有一层特定的吸附剂作为固相萃取相^［31］^。在正式操作前，需根据分析需求精心选择萃取相（包括涂层类型和厚度），并按要求对其进行活化或老化等预处理。常用的涂层材料包括：聚二甲基硅氧烷（PDMS）、聚丙烯酸酯（PA），以及聚二甲基硅氧烷/聚二乙烯基苯（PDMS-DVB）、碳纤维/聚二乙烯基苯（CW-DVB）、碳纤维/分子印迹树脂（CW-TPR）等混合材料^［32］^。随后，将萃取相浸入待测样品中或放置在样品上方，确保萃取相与样品充分接触。萃取过程中的参数，如萃取时间、温度和搅拌速度等，均可根据具体实验需求进行精确调整和优化。在萃取阶段，目标分析物会从样品基质中逐渐转移到萃取相涂层上，实现其吸附和富集。萃取完成后，将SPME纤维从样品中取出并插入到分析仪器中，如GC或HPLC中。SPME萃取和解吸样品的基本流程如图2 ^［33］^所示。在高温或溶剂的作用下，目标分析物从萃取涂层上解吸下来，并被仪器检测和分析^［33-35］^。

**图2:**
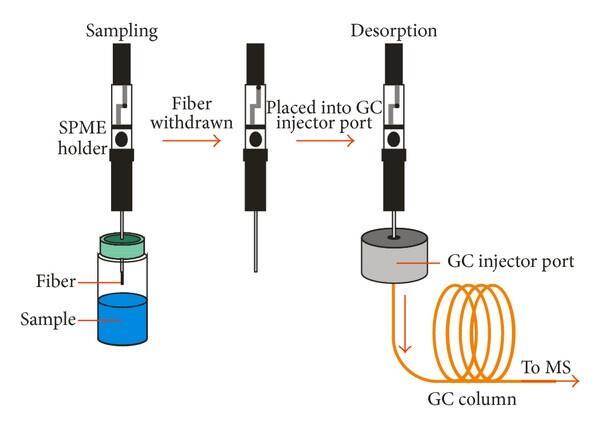
用SPME-GC-MS萃取和解吸样品的流程^［33］^

SPME技术具有多种操作模式，包括传统的涂层纤维式、管内固相微萃取（IT-SPME）、搅拌棒吸附萃取（SBSE）、分散固相微萃取（DSPME）、薄膜微萃取（TFME）、尖端固相微萃取以及针式固相微萃取等^［36］^。这些模式为不同样品的分析提供了灵活的选择，拓展了SPME技术的应用范围。

### 1.2 MIPs的制备

MIPs是一种仿生识别材料，采用MIT，通过模拟生物分子识别机制，为特定目标分子构建具有“锁-钥”匹配结构的人工受体。MIT利用模板分子引导合成具有特异性结合位点的聚合物，实现对目标分子的高选择性识别^［37-39］^。

#### 1.2.1 MIPs的聚合方法

近5年来，报道的复杂样品中药物残留检测所使用的MI-SPME中，MIPs的制备方法主要有自由基聚合法、原位聚合法和溶胶-凝胶法^［40，41］^。

自由基聚合法 自由基聚合法是合成MIPs的重要方法，主要包括本体聚合、沉淀聚合、乳液聚合、悬浮聚合和可逆加成-断裂链转移（RAFT）聚合等多种形式^［42］^。其中，本体聚合是最简单直接的方法，单体在无溶剂条件或其他分散介质下通过引发剂、光或热的作用直接聚合，所得产物纯度高^［43，44］^，但需要后续研磨以获得合适粒径。沉淀聚合则通过将单体和引发剂溶于适当溶剂，使聚合物以沉淀形式析出，无需后续研磨即可获得粒径均匀的MIPs，操作简便且产物易分离^［45，46］^。乳液聚合在乳液体系中进行，具有反应条件温和、产物分散性好的特点，特别适用于复杂基质中目标分子的鉴定^［47-49］^。悬浮聚合法以水为连续相，通过稳定剂或表面活性剂将预聚合混合物乳化成悬浮液滴，能够有效控制产物粒径^［50-52］^。RAFT聚合通过引入链转移剂实现对聚合物分子质量和结构的精确调控^［51］^，可应用于多种聚合体系^［53］^。

原位聚合法 原位聚合法是目前制备MI-SPME装置的主要方法，是一种直接在载体表面进行聚合的技术，通过将预组装溶液涂覆或注入载体，经聚合和模板去除后形成具有特定空穴结构的MIPs。该方法避免了后续研磨步骤，能有效保持材料的识别位点完整性。Chen等^［54］^通过原位聚合法合成了一种基于硼氮配位相互作用的MIPs，用于豆浆和葡萄汁样品中磺酰脲类除草剂（SUHs）的SPME。

溶胶-凝胶聚合法 溶胶-凝胶法是一种温和的水相合成技术，通过前驱体水解缩合形成三维网络结构来制备MIPs。其克服了传统有机相合成的局限性，具有更好的水相识别能力和环境友好性^［55，56］^。例如，Moeini等^［57］^基于溶胶-凝胶法，通过静电纺丝技术在不锈钢棒表面制备MIPs纳米纤维，开发了一种高稳定性的在线SPME工具，成功用于饮料中乙酰磺胺酸的高选择性在线测定。

### 1.3 新的印迹技术与印迹策略

传统MIPs存在模板泄漏、结合能力有限、材料形状不规则以及在水溶液中不相容性等缺陷。为解决这些问题，并进一步针对不同目标分子的特性和复杂样品基质的需求，新的印迹技术与印迹策略应运而生，显著提升了MIPs的性能。印迹技术指MIPs的具体制备方法，印迹策略侧重功能设计与应用思路，二者相辅相成。目前，这些技术与策略已广泛用于MI-SPME装置，实现了复杂样品中痕量药物的高效、精准检测，主要包括以下几类：表面印迹技术、纳米印迹技术、虚拟印迹策略、多模板/功能单体印迹策略以及刺激响应印迹策略等^［38］^。

#### 1.3.1 表面印迹技术

表面印迹技术指通过在材料（如碳纳米管（CNTs）、Fe_3_O_4_、SiO_2_和聚四氟乙烯（PTFE）膜等）表面或近表面区域制备薄层聚合物材料，构建高效识别位点^［38］^，能够解决传统MIPs结合能力低和洗脱困难等问题。其中，核壳结构因其高比表面积和强结合能力成为表面印迹MIPs的主要类型^［42］^。表面印迹技术能有效增加MIPs识别位点的可及性，加快传质速率，适于MI-SPME。例如，Jian等^［58］^以2，4-D为模板，4-乙烯基吡啶为单体，PTFE为基底，采用表面印迹技术制备了MI-SPME涂层，成功用于牛奶中2，4-D的选择性萃取与检测。

#### 1.3.2 纳米印迹技术

纳米印迹技术是通过制备纳米级MIPs，利用其高比表面积特性暴露更多结合位点，显著提升对目标分析物的结合能力^［59］^。该技术通常结合沉淀聚合、溶胶-凝胶聚合^［60］^来获得纳米MIPs，并可根据材料维度分为零维（纳米颗粒）、一维（纳米纤维/管）和二维（石墨烯）等类型^［18］^。Ma等^［61］^利用纳米印迹技术将分子印迹纳米聚合物聚合到金属有机骨架（MOFs）材料表面，并应用于鸡肌肉组织样品中7种TCs的SPME。

#### 1.3.3 虚拟模板印迹策略

虚拟模板印迹策略是采用与目标化合物结构和功能相似的稳定化合物作为模板^［40，62］^以制备虚拟MIPs，适用于难获取、成本较高、易降解或在聚合条件下不稳定的靶标分析物^［46］^。该印迹策略避免了模板泄漏问题，提高了检测结果的准确性。Marchioni等^［63］^利用虚拟模板印迹策略，以氢化大麻二酚（CBD）为虚拟模板，在熔融石英毛细管中原位聚合制备MIPs。发展了一种新型MI-SPME，成功用于患者血浆中大麻素的治疗监测。

#### 1.3.4 多功能单体印迹策略

多功能单体印迹策略是利用两种及以上功能单体与模板分子相互作用^［64］^，从而优化识别和结合效果^［65］^，已被广泛用于药物残留检测等领域中。Dowlatshah等^［66］^采用多功能单体印迹策略，以（3-氨基丙基）三乙氧基硅烷（APTES）和苯基三甲氧基硅烷（PTMOS）为功能单体，共同与苯醚甲环唑模板作用，制备了一种基于二氧化硅的多模板MIPs，作为SPME吸附剂，高效萃取了小麦和水果样品中的苯醚甲环唑。

#### 1.3.5 多模板印迹策略

多模板印迹策略是通过同时使用两种及以上模板分子，在单一材料中构建多种识别位点^［67］^，可同时识别和富集多种分析物，克服了单模板MIPs的局限性和提高了MIPs的使用效率。Luo等^［68］^使用多模板印迹策略，以4种肝毒性吡咯里西啶生物碱（HPA）为模板，制备了MI-SPME纤维，用于香茶中10种HPA的选择性萃取富集，为复杂样品多目标分析提供了新材料和新方法。

#### 1.3.6 刺激响应型印迹策略

刺激响应分子印迹策略是通过引入pH、光、热或磁等响应性单体，利用活性聚合或点击化学等技术制备刺激响应型印迹聚合物（SR-MIPs）。在外界刺激条件下，MIPs的特性会发生显著可逆变化，如溶解度、表面结构、分子链结构、溶蚀或解离行为等^［69］^。从而实现对目标分子的智能识别与可控释放^［70］^。例如，Dil等^［71］^开发了一种基于Fe₃O₄@SiO₂-MIPs的SPME技术，用于水和生物样本中褪黑激素（MLT）的提取。该磁性MIPs（MMIPs）材料具有快速磁分离能力、优异的重复使用性，且对MLT表现出高选择性。郭跃龙等^［72］^合成了一种热响应型可修复MI-SPME纤维，以硅烷化石英毛细管为载体，成功用于蜂蜜样品中痕量大环内酯类抗生素（MACs）残留的高效分析。通过调控温度，可实现材料的智能吸附与脱附，提高检测效率。Ansari等^［73］^制备了一种pH敏感型MIPs，结合SPME，实现了生物体液中抗癌药物卡培他滨（CAP）的高选择性萃取。该材料在特定pH条件下表现出可调控的吸附与释放行为，提高了分析方法的选择性和回收率。

### 1.4 MIPs结构设计与目标分子适配策略

MIPs的特异性识别能力高度依赖其识别位点与目标分子结构和官能团的精确匹配。为实现这一目标，需根据目标分子的化学性质（如官能团、极性、空间构型）优化功能单体和交联剂的选择与配比^［22］^。例如，对于含羧基/羟基的极性药物（如2，4-D、萘普生），可选用4-乙烯基吡啶（4-VP）或甲基丙烯酸（MAA）等兼具氢键供体/受体特性的功能单体，通过非共价相互作用（如氢键或静电作用）提升识别效率。Jian等^［58］^研究表明，针对2，4-D的MIPs通过4-VP与羧基的氢键相互作用可实现特异性识别。对于含杂环或金属配位位点的药物（如TCs），可采用金属配位单体或多功能单体协同策略，如Lu等^［74］^通过MAA与甲基丙烯酸2-羟乙基（HEMA）双功能单体证实其能协同识别TCs的酮基和羟基位点，从而显著增强选择性。

## 2 MI-SPME模式

SPME的萃取模式主要依赖于基体支持物（如石英纤维、不锈钢丝等）表面固定相与目标分析物之间的相互作用。MIPs以其高选择性为优势，在与SPME结合后，能够显著提高萃取的选择性和效率。为了满足不同待测样品的特性和检测仪器的要求，MI-SPME介质需经化学、物理手段设计成适配的技术模式以便在实际样品的分离、富集及测定过程中得到应用。在药物分析领域，MI-SPME的主要模式有MIPs涂层纤维SPME、基于MIPs的IT-SPME、MIPs-SBSE、MIPs-DSPME、MIPs-TFME和MIPs尖端SPME^［75］^。

### 2.1 MIPs涂层纤维SPME

该模式以涂覆MIPs的石英纤维或不锈钢丝作为吸附介质，凭借操作直观简便的特性成为MI-SPME的基础形式，其萃取机制依赖于目标物在样品基质与涂层间的扩散和分配平衡。操作方式主要包括直接浸入式和顶空式。直接浸入式是将纤维浸入液体样品中（尤其适配中小分子、中等极性目标物如抗生素），通过直接接触实现富集。该方法无需使用有机溶剂，操作简便且环保^［76，77］^；顶空式则是将纤维置于样品上方的顶空区域，适用于挥发性/半挥发性目标物，这种方式能够有效避免复杂基质的干扰，从而提升对目标物的选择性^［78，79］^。然而，受限于涂层比表面积和厚度，该模式的吸附容量相对较低，且涂层直接暴露易因机械摩擦而磨损，使用寿命短，因此更适用于自动化需求低的小规模分析及中等体积样品^［34］^。目前，直接浸入式SPME在药物分析领域应用广泛。例如，Li等^［80］^采用沉淀聚合法构建了基于共价有机框架（COFs）MIPs（CMIPs）涂层的SPME纤维阵列，有效提取了牛奶和蜂蜜样品中的硫酸妥布霉素（TOB）。

### 2.2 基于MIPs的IT-SPME

IT-SPME因其微型化、自动化以及与色谱/质谱的良好兼容性而备受关注，主要包括涂层式、整体柱式和填充柱式3种类型^［81-83］^。该技术以毛细管为萃取装置，具有传质路径短、萃取动力学快、样品消耗少的核心优势，特别适用于液体样品（尤其是水样、生物体液）中痕量目标物的高通量自动化在线分析，在复杂基质样品的抗干扰萃取中表现突出。不过，其也存在一定局限，例如对非极性目标物的萃取能力相对较弱，且毛细管内径较小可能导致样品处理量受限^［84］^。在药物分析领域，MIPs与IT-SPME的结合展现出显著优势，目前应用较广泛的主要是涂层式和整体柱式IT-SPME。

#### 2.2.1 MIPs涂层式IT-SPME

MIPs涂层式IT-SPME，是指将MIPs材料采用物理涂覆或化学键合方式在不同材质的毛细管内壁上制备萃取涂层，以此获得具备特定萃取功能的毛细管柱^［84］^。例如，Kefayati等^［85］^采用分子印迹聚吡咯与氧化铜的纳米复合材料（MIP@CuO）作为IT-SPME的涂层，成功实现了尿液和血浆样品中卡马西平（CBZ）的高效萃取，不仅提高了选择性，同时简化了样品前处理流程。

#### 2.2.2 基于MIPs的整体柱式IT-SPME

基于MIPs的整体柱式IT-SPME，是指利用原位聚合、溶胶-凝胶法等手段，在毛细管内直接合成MIPs整体柱。该整体柱具有双连续结构，即形成贯穿大孔与连通中孔并存的双孔分布体系。和传统填充柱相比，整体柱没有颗粒间的间隙，通透性更佳^［86］^，合成过程更简便，萃取容量更大，稳定性和使用寿命都有一定提升^［63，87］^。Zhou等^［88］^以E2为模板分子，采用原位聚合法构建了整体柱式MI-SPME纤维阵列，为复杂基质中雌激素残留分析提供了一种高效、稳定的前处理方法。

### 2.3 MIPs-SBSE

MIPs-SBSE是一种集无溶剂萃取与高容量富集于一体的样品制备技术，其核心是以磁力搅拌棒为载体，通过将MIPs介质材料涂覆于搅拌棒表面，在持续搅拌过程中实现对目标物的高效萃取与富集^［89］^。此外，由于搅拌棒的表面积较大，该技术具有显著高于传统纤维SPME的吸附容量，尤其适用于痕量分析的高富集需求。在药物分析领域，MIPs-SBSE已获得广泛应用^［90］^。例如，Tang等^［91］^制备了一种新型MIPs涂层搅拌棒，同时提取6种*β*-激动剂，为动物源食品中*β*-激动剂残留测定提供了一种强有力工具。

### 2.4 MIPs-DSPME

MIPs-DSPME是一种简单、快速、经济的方法。通过将MIPs作为吸附剂直接分散到样品溶液中，借助磁力搅拌或超声辅助实现高效萃取。该方法的显著优势在于吸附剂与样品溶液的充分接触，不仅显著提高了萃取效率和富集倍数，还减少了样品消耗量，缩短操作时间，特别适用于复杂固体/半固体样品（如肉类、组织、土壤）提取液的快速处理^［92］^。目前，MIPs-DSPME在药物分析领域得到了广泛应用。例如，Bazmandegan等^［93］^建立了MMIPs-DSPME方法，对食品和水样中的杀螟硫磷（FNT）具有较高的吸附能力和选择性。

### 2.5 MIPs-TFME

TFME因其出色的复杂基质处理能力而备受关注。与涂层纤维式SPME和IT-SPME相比，该技术采用薄层大体积吸附剂设计，通过增大有效表面积与体积比，在保持优异传质动力学的同时显著提升了萃取效率，且能快速达到吸附平衡^［94，95］^。这种结构特性使其对复杂基质具有出色的处理能力。因此TFME适用于需要兼顾抗基质干扰能力和快速萃取的场景^［96］^。由于配方和制造的灵活性，MIPs成为TFME的理想吸附材料，既可作为固体基底支撑薄膜，也可制成独立薄膜使用。例如，Fazli等^［97］^在不锈钢上制备了一种新型导电MIPs薄膜，成功用作电化学控制SPME的选择性吸附剂，用于分析作为麻醉药物的硫喷妥钠（TP），为复杂基质中的TP分析提供了有效方法。

### 2.6 MIPs尖端SPME

MIPs尖端SPME技术通过在移液管尖端填充或涂覆MIPs材料，实现对目标分析物的选择性萃取。当样品溶液被吸入尖端时，目标分析物与MIPs上的特定识别位点相互作用，而其他干扰物质则被有效排除，从而实现目标分析物的萃取和分离^［98，99］^。该技术将样品前处理过程直接整合到移液操作中，无需复杂的分离装置，显著提高了实验效率，特别适合微量样品的快速分析。例如，Arabi等^［100］^通过原位聚合法在移液管尖端直接合成了MIPs涂层，并成功将该技术应用于橙汁样品中痕量没食子酸的高选择性检测。

## 3 MI-SPME结合色谱/质谱在药物残留测定中的应用

药物残留测定在确保食品安全、用药安全、预防环境污染及维护公众健康等方面具有重要意义^［101］^。MI-SPME具有溶剂消耗少、样品处理高效等独特优势^［25］^，结合色谱/质谱，已广泛应用于食品、环境及生物样品中的药物残留检测。表1~3分别汇总了不同MI-SPME装置在食品、环境和生物样品中的典型应用，以下将分领域展开分析。

**表1:** MI-SPME结合色谱/质谱在食品样品药物残留测定中的应用

Analytes	Template	Polymerizationmethod	Imprinting technique and strategy	SPME mode	Chromatography/MS	Linear range	LOD	Recovery/%	Real samples	Ref.
Five MACs	ROX	-	surface imprinting	in-tip SPME	ESI-MS	0.05-100 ng/g	0.003-0.005 ng/g	73.4-98.1	drinking water， honey， milk	［^102^］
Four MACs	piramycin	-	stimuli-responsive imprinting	coated fiber SPME	HPLC-UV	0.5-50 μg/mL	23.8-85.4 μg/kg	81.8-119.1	honey	［^72^］
Four SAs	SMM	emulsion polymerization	surface imprinting	SBSE	HPLC-MS/MS	10-1000 μg/L	1.5-3.4 μg/L	80-89	animal feeds	［^49^］
Four SAs	silver sulfadiazine	-	nano-imprinting	TFME	HPLC-UV	0.01-50 μg/L	0.003 μg/L	95.9-101.0	milk， eggs and chicken meat	［^103^］
Seven TCs	minocycline	-	nano-imprinting	DSPME	UHPLC-PDA	0.5-200 ng/mL	0.2-0.6 ng/g	69.6-94.7	chicken muscle	［^61^］
Five TCs	TC	bulk polymerization	multi-functional monomer imprinting	coated fiber SPME	HPLC-UV	5-1000 μg/L	0.38-0.72 μg/kg	77.3-104.4	milk， chicken， fish	［^74^］
Six aminoglycosides	TOB	precipitation polymerization	-	coated fiber SPME	UHPLC-MS/MS	0.1-500 μg/kg	0.002-0.02 μg/kg	86.2-115.3	milk， honey	［^80^］
Five estrogens	E2	in situ polymerization	-	IT-SPME	HPLC-DAD	1.00-200.00 μg/L	0.33 μg/L	74.75-119.41	milk， yogurt	［^88^］
E2	E2	in situ polymerization	-	IT-SPME	HPLC-UV	0.10-200.00 μg/kg	0.03 μg/kg	79.61-105.70	meat	［^104^］
Seven BZs	2-aminobenzimidazole	-	dummy template imprinting	DSPME	UHPLC-PDA	5-2000 ng/mL	0.2-0.5 ng/g	92.3-97.1	beef	［^105^］
*β*-Agonist	clenbuterol	-	surface imprinting	SBSE	HPLC-MS/MS	0.5-35 μg/L	0.05-0.15 μg/kg	75.8-97.9	pork	［^91^］
2，4-D	2，4-D	-	surface imprinting	TFME	HPLC-UV	1.0-10 mg/L	0.03 mg/L	88.8-96.6	milk	［^58^］
Six neonicotinoids	CLT	-	-	IT-SPME	HPLC-DAD	50-4000 μg/L	0.03-0.58 μg/L	85.4-116.8	tea， honey	［^106^］
Four pyrethroids	enpropathrin， deltamethrin， cyfluthrin， bifenthrin	-	surface imprinting	coated fiber SPME	HPLC-DAD	0.50-200.00 μg/L	0.16-0.33 μg/L	72.74-119.66	tea	［^107^］
Difenoconazole	difenoconazole	sol-gel method	surface imprinting	coated fiber SPME	GC-ECD	0.01-1 ng/mL	0.002 ng/mL	73-103	wheat， cucumber， apple	［^66^］
Five SUHs	triflusulfuron-methyl	in situ polymerization	-	IT-SPME	HPLC-DAD	0.10-200.0 μg/L	0.014-0.058 μg/L	75.2-102	spiked soya milk， grape juice	［^54^］
Five OPPs	diazinon， parathion-methyl， isocarbophos	sol-gel method	multi-template imprinted	coated fiber SPME	GC-NPD	0.1-100 μg/kg	0.0052-0.23 μg/kg	75.1-123.2	spiked apple， cucumber， Chinese cabbage， cherry tomato	［^28^］
Five OPPs	diazinon， isocarbophos	sol-gel method	-	coated fiber SPME	GC-NPD	0.1-1000 μg/kg	0.0028-0.0436 μg/kg	78.7-122.8	apple， potato	［^108^］
Five OPPs	ethion	-	stimuli-responsive imprinting	DSPME	GC-FID	0.50-2000 μg/L	0.25-0.50 μg/L	93-117	fruit， vegetable	［^109^］
Pyriproxyfen	pyriproxyfen	sol-gel method	multi-functional monomer imprinting	DSPME	HPLC-DAD	5.25×10^-5^-43 μg/mL	4.93×10^-5^ μg/mL	95.9-97	strawberry	［^110^］
Thiabendazole and carbendazim	thiabendazole	sol-gel method	surface imprinting	SBSE	HPLC-DAD	25-1000 μg/L	0.10-0.13 mg/kg	21-33	orange	［^111^］
Paraquat	paraquat	sol-gel method	-	SBSE	HPLC-UV	0.02-0.85 mg kg	0.005 mg/kg	70.0-96.1	lettuce	［^112^］
Fipronil	fipronil	precipitation polymerization	-	DSPME	HPLC-DAD	6×10^-3^-45 μg/mL	5.64×10^-6^ μg/mL	94.6-96.5	milk	［^113^］
Quercetin	quercetin	sol-gel method	surface imprinting	coated fiber SPME	HPLC-UV	0.05-100 μg/mL	9.94 ng/mL	94.20-98.50	tea， coffees	［^114^］
Four amphetamine derivatives	dextroamphetamine	-	nano-imprinting	IT-SPME	GC-MS	0.1-400 μg/L	0.023-0.033 μg/L	96.2-98.9	beverage， snack	［^115^］
Amphetamines and modafinil	amphetamines， modafinil	-	-	IT-SPME	GC-MS	0.1-400 μg/L	0.023-0.033 μg/L	95.14-104.63	medicinal supplements	［^116^］

MACs： macrolide antibiotics； ROX： roxithromycin； SAs： sulfonamides； SMM： sulfamonomethoxine； TC： tetracycline； E2： estradiol； BZs： benzimidazoles； 2，4-D： 2，4-dichlorophenoxyacetic acid； CLT： clothianidin； SUHs： sulfonylurea herbicides； OPPs： organophosphorus pesticides； CBZ： carbendazim； TOB： tobramycin； SBSE： stir bar sorptive extraction； TFME： thin-film microextraction； DSPME： dispersive solid-phase microextraction； IT-SPME： in-tube solid-phase microextraction.

**表2:** MI-SPME结合色谱/质谱在环境样品药物残留测定中的应用

Analytes	Template	Polymerization method	Imprinting technique and strategy	SPME mode	Chromatography/MS	Linear range	LOD	Recovery/%	Real samples	Ref.
Four triazines	propazine	-	dummy template imprinting	IT-SPME	HPLC-DAD	100-1000 μg/L	6.2-15.7 ng/g	75.7-120.1	soil	［^117^］
Sulfamethoxazole	sulfamethoxazole	-	nano-imprinting	DSPME	HPLC-UV	7-900 ng/mL	2.0 ng/mL	94.2-98.2	spiked water	［^118^］
Four FQs	ENRO	-	-	IT-SPME	HPLC-UV	10-500 μg/L	0.1-10 μg/L	9.4-24.5	surface water， groundwater， urine	［^119^］
NOR	NOR	precipitation polymerization	surface imprinting	TFME	HPLC-DAD	-	0.15 μg/L	90.1-102.7	seawater， fish	［^120^］
Phenobarbital	phenobarbital	sol-gel method	surface imprinting	coated fiber SPME	HPLC-UV	0.01-4 μg/mL	7.5 ng/mL	92.4-98.0	spiked river， well water	［^121^］
Aniline	aniline	suspension polymerizatio	surface imprinting	DSPME	HPLC-MS	1-200 ng/mL	1 ng/mL	62	textile wastewater	［^52^］
Diazepam	diazepam	bulk polymerization	-	SBSE	UHPLC-MS/MS	0.05-500 μg/L	0.4 ng/L	84-102	natural water	［^122^］
Naproxen	s-naproxen	-	-	SBSE	HPLC-DAD	0.01-200 μg/L	0.005 μg/L	83.98-118.88	lake water， river water	［^123^］

FQs： fluoroquinolones； ENRO： enrofloxacin； NOR： norfloxacin.

**表3:** MI-SPME结合色谱/质谱在生物样品药物残留测定中的应用

Analytes	Template	Polymerization method	Imprinting technique and strategy	SPME mode	Chromatography/MS	Linear range	LOD	Recovery/%	Real samples	Ref.
TP	TP	-	surface imprinting	TFME	IMS	3.3-200 μmol/L	1.1 μmol/L	81-94	human serum	［^97^］
Chrysophanol	chrysophanol	radical polymerization	surface imprinting	coated fiber SPME	HPLC-UV	0.007-0.2 μg/mL	2.68 ng/mL	94.01-96.20	urine	［^124^］
Phenobarbital	phenobarbital	sol-gel method	surface imprinting	coated fiber SPME	HPLC-UV	0.02-100 μg/mL	9.88 ng/mL	94.26-98.50	urine	［^27^］
Propranolol	propranolol	RAFT polymerization	-	DSPME	HPLC-UV	0.015-100 μmol/L	0.002 μmol/L	85.2-97.4	bovine serum	［^53^］
CBZ	CBZ	in situ polymerization	nano-imprinting	IT-SPME	HPLC-UV	0.01-500 μg/L	0.05 μg/L	92.0-114.0	urine， plasma	［^85^］
OXY	OXY	-	stimuli-responsive imprinting	DSPME	HPLC-UV	1-2000 ng/mL	0.80 ng/mL	92.50-103.20	urine	［^125^］
Harmaline	harmaline	-	stimuli-responsive imprinting	DSPME	HPLC-UV	1.0-4000 ng/mL	0.526 ng/mL	90.00-99.25	peganum harmala	［^126^］
Hymol and carvacrol	hymol， carvacrol	-	stimuli-responsive imprinting	DSPME	HPLC-UV	0.40-5000 ng/mL	0.042 ng/mL	96.6-105.4	summer savoury， *Origanum* majorana， *Origanum* vulgare	［^127^］
CAP	CAP	RAFT polymerization	stimuli-responsive imprinting	DSPME	HPLC-UV	5-2000 ng/mL	1.9 ng/mL	93.41-102.50	plasma	［^73^］
MLT	MLT	-	stimuli-responsive imprinting	DSPME	HPLC-UV	0.2-4200 ng/mL	0.046 ng/mL	93.07-104.1	urine， plasma	［^71^］
CBD	CBD	in situ polymerization	-	IT-SPME	UHPLC-MS/MS	10-300 ng/mL	-	53	plasma	［^63^］
Digoxin	digoxin	in situ polymerization	surface imprinting	coated fiber SPME	HPLC-UV	0.1-10 ng/mL	0.03 ng/mL	-	urine， serum	［^128^］
Valproic acid	valproic acid	-	-	coated fiber SPME	GC-FID	0.03-100 μg/L	0.01 μg/L	90.5-7.5	human serum	［^129^］

TP： thiopental； CBZ： carbamazepine； OXY： oxycodone； CAP： capecitabine； MLT： melatonin； CBD： cannabidiol； RAFT polymerization： reversible addition-fragmentation chain-transfer polymerization.

### 3.1 食品安全

近年来，MI-SPME技术在食品安全领域，特别是在牛奶、水果等多种食品样品的药物残留检测方面发挥了重要作用。借助MI-SPME，能够高效且准确地检测出食品中的药物残留，从而确保食品的安全性。目前，该技术主要应用于食品样品中兽药残留^［49，61，72，74，80，88，91，102-105］^、农药残留^［28，54，58，66，106-113］^和人用药物^［114，115］^的检测。

Lu等^［74］^采用多功能单体印迹策略，以TC为模板，以MAA和甲基丙烯酸2-羟乙基（HEMA）为双功能单体，采用改进的多重共聚方法，将MIPs固定在不锈钢丝上，合成了一种兼具亲水性和选择性的MI-SPME。该纤维可直接与HPLC-UV联用，用于动物源性食品（牛奶、鸡肉和鱼类）中TCs残留的灵敏测定。结果表明，TCs的线性范围为5~1 000 μg/L，检出限（LOD）为0.38~0.72 μg/kg，回收率为77.3%~104.4%。该纤维具有良好的选择性和重复性，相对标准偏差（RSD）为6.2%~7.8%。该研究表明，这种绿色与水兼容的MI-SPME纤维在复杂基质痕量TCs的高效识别与分离方面具有广阔的应用前景。

Liu等^［102］^建立了一种新型的ESI-MS方法，用于复杂食品样品中痕量大环内酯（MACs）的灵敏分析。以罗红霉素（ROX）为模板，采用表面印迹技术在木质针尖上原位合成MIPs涂层，成功制备了一种新型MI-SPME纤维。结合ESI-MS法，对饮用水和蜂蜜、牛奶等复杂食品样品中的5种目标MACs进行了高选择性、高灵敏度的萃取。结果表明，富集因子（EF）分别为244~1 604、72~370和12~82倍。LOD为0.003~0.005 ng/g，加标回收率为73.4%~98.1%，RSD≤8.6%。为饮用水和复杂食品底物中微量抗生素的检测提供了新方法。

Cui等^［49］^开发了一种基于分子印迹静电纺纳米纤维膜（MIM）的SBSE方法，用于快速测定动物饲料样品中的磺胺类药物（SAs）。首先，以磺胺间甲氧嘧啶（SMM）为模板、MAA为功能单体、乙二醇二甲基丙烯酸酯（EGDMA）为交联剂，通过乳液聚合法制备了MIPs。然后，利用静电纺丝技术，将掺杂了MIPs的静电纺丝溶液制成具有可控形态的MIM。所制备的MIM被涂覆在磁搅拌棒表面，可在HPLC-MS/MS分析前直接用于提取饲料样品中的SAs。结果表明，所得到的MIM对SAs具有良好的类别选择性（选择性因子为2.3~2.7）、良好的再生能力和可重复使用性。该方法的LOD为1.5~3.4 ng/g，实际样品回收率为80%~89%。因此，该方法在不同动物饲料中痕量污染物的快速分离富集方面具有很大潜力，为食品安全检测提供了一种高效、可靠的新方法。

Mirzajani和Kha^［103］^以磺胺嘧啶银为模板分子、MAA为功能单体、EGDMA为交联剂、偶氮二异丁腈（AIBN）为引发剂，制备了基于双金属MOF@高岭土/深共晶溶剂/分子印迹复合材料（Co_0.5_Zn_0.5_（MeIm）_2_@HNT/DES/MIPs复合材料）的电纺丝薄膜纳米纤维。将其应用于TFME技术，结合HPLC-UV，实现了牛奶、鸡蛋和鸡肉中4种SAs残留的灵敏检测。结果表明，该薄膜在0.01~50 μg/L的线性范围内表现良好，LOD为0.003 μg/L，LOQ为0.01 μg/L。该方法具有操作简单、制样成本低、萃取效率高、基质干扰小等优点。因此，该研究为SAs残留检测提供了一种高效、灵敏、经济的方法，有助于确保食品供应链的纯度和安全性。

Zhang等^［104］^基于适配体和分子印迹的双重识别策略，以E2为模板分子、MAA为功能单体，采用原位聚合法制备得到适配体-分子印迹（Apt-MIP），该材料对E2具有显著的特异性识别和富集能力，EF为187.58。基于Apt-MIP的IT-SPME结合HPLC-UV，测定猪肉、鸡肉、鱼、虾等样品中的E2，回收率为79.61%~105.70%，LOD为0.03 μg/kg；Apt-MIP纤维涂层具有良好的稳定性，可重复使用15次以上。本研究为基于MIT的样品前处理技术提供了新的视角和策略，提高了分析性能。

Wang等^［105］^采用虚拟模板印迹策略在氮化硼表面合成了MIPs微球，并将其作为吸附剂，建立了MIPs-DSPME方法，用于萃取牛肉中的苯并咪唑类药物（BZs），随后通过UHPLC-PDA进行测定。该MIPs材料可同时识别7种BZs，具有高吸收率（36.23~38.88 μg/mg），可重复使用5次。该MIPs-DSPME-UHPLC-PDA方法对7种BZs具有较高的EFs（21.5~37.9），在牛肉样品中的LOD和LOQ分别在0.2~0.5 ng/g和0.5~1.5 ng/g范围内，回收率高（92.3%~97.1%）。因此，该方法可用于肉类样品中BZs残留的常规测定，为食品安全监管提供可靠的技术支撑。

Seebunrueng等^［109］^采用刺激响应印迹策略，以混合价态氢氧化铁纳米颗粒作为磁性载体，*N*-异丙基丙烯酰胺作为热敏单体、乙硫磷作为模板、MAA作为功能单体进行聚合反应，制得了一种新型热敏磁性分子印迹聚合物（TMMIP）。将其用作DSPME的吸附剂，通过GC-FID测定苹果、橘子、葡萄、番石榴、红辣椒、番茄、黄瓜和豆角样品中的5种痕量OPPs。该方法具有良好的线性关系（0.50~2 000 μg/L）、低LOD（0.25~0.50 μg/L）、低LOQ（0.50~1.50 μg/L）以及高精度（RSD<7%），回收率为93%~117%，实现了对多种果蔬样品中OPPs农药残留的准确、高效测定。这项研究表明所开发方法在确保食品安全和质量控制方面具有重要的实际应用价值，助力保障公众健康和消费者福祉。

Wang等^［106］^采用自由基聚合技术制备了一种独特的MI-SPME纤维阵列，以氯噻嗪（CLT）为模板分子、MAA为功能单体、乙二醇二甲基丙烯酸酯（EDMA）为交联剂、AIBN为引发剂，在玻璃毛细管中合成CLT-MIPs，将其作为IT-SPME的吸附剂。将该纤维阵列与HPLC-UV联用，用于筛选食品样品（茶叶和蜂蜜）中新烟碱类农药的残留。结果表明，与两种商业纤维阵列相比，CLT-MIP 纤维阵列对6种新烟碱类化合物的富集倍数显著提高了1 189~2 356倍。此外，CLT-MIP纤维阵列还表现出良好的重现性和可重复使用性；至少可重复使用100次，且吸附性能不明显下降。LOD为0.03~0.58 μg/L，回收率为85.4%~116.8%。这种印迹纤维阵列具有高通量、重现性好以及多组分定量准确的优点，为高效富集和高通量同时检测食品样品中的多种化合物开辟了新途径，提高了食品中农药残留检测的效率和准确性。

Kardani等^［115］^以右旋安非他命为模板分子，开发了一种基于氧化石墨烯（GO）@ZIF-8 MOF/MIP复合材料的整体式IT-SPME方法，结合GC-MS技术，实现了对饮料和零食中非法添加的安非他命衍生物的高选择性富集和检测。该方法LOD低至0.023~0.033 μg/L，回收率大于96.2%，充分证明了其准确性和可靠性。相较于传统方法，这类基于GO@ZIF-8 MOF/MIP的IT-SPME具有选择性强、操作简单、成本效益高等优点，能够有效辨识膳食补充剂中的掺假行为，为食品安全监管提供有力技术支撑，有助于保障消费者的健康与安全。

Hayat等^［110］^采用多功能单体印迹策略，以吡丙醚为模板分子、丙烯酰胺（AA）和3-（三甲氧基硅基）甲基丙烯酸丙酯（TMSPM）为功能单体，采用溶胶-凝胶法合成分子印迹有机改性二氧化硅（MIormosil）。以MIormosil为DSPME吸附剂，结合HPLC-DAD测定草莓样品中的吡丙醚。结果表明，该方法的LOD和LOQ分别为4.93×10^-5^ μg/mL和1.49×10^-4^ μg/mL。MIormosil对吡丙醚的最大结合容量和回收率分别为13 mg/g（*n*=5）和97.3%，可重复使用至少5次。因此，该基于MIormosil的DSPME方法为草莓等食品中吡丙醚残留的高效、准确检测提供了新的前处理方法，有望在保障食品安全方面发挥重要作用。

Jian等^［58］^采用表面印迹技术，2，4-D为模板、4-VP为功能单体、PTFE膜为支撑材料，制备了新型的MI-SPME涂层，用于牛奶样品中2，4-D的萃取。该MI-SPME涂层对2，4-D的吸附容量达41.8 mg/g。经137次再生使用后仍表现出优异的结构稳定性和重复使用性，RSD≤6.35%。所建立的基于MIPs的TFME-HPLC-UV可用于牛奶样品中2，4-D的选择性提取和测定，回收率为88.8%~96.6% （RSD≤5.1%），LOD为0.03 mg/L，LOQ为0.1 mg/L。因此，该MIPs材料制备简单，成本低，寿命长，有望为食品样品中其他种类植物激素富集提供有效的SPME新涂层。

Dowlatshah等^［66］^采用多功能单体印迹策略，以APTES和PTMOS为功能单体，基于表面印迹技术和溶胶-凝胶法，在介孔二氧化硅（MCM-41）表面构建了三维网络分子印迹涂层，成功制备了MIPs涂层纤维式SPME，结合GC-ECD，实现了对苯醚甲环唑的高选择性萃取和测定。该方法表现出优异的分析性能，LOD低至0.002 ng/mL，LOQ为0.005 ng/mL，在0.01~1 ng/mL范围内呈良好线性，RSD小于10%。该MIPs涂层纤维式SPME成功应用于小麦和水果样品中苯醚甲环唑的萃取，为食品安全监测领域提供了一种新型、高效的分析工具。

在食品安全领域，MI-SPME技术凭借MIPs的高选择性与SPME的高效富集能力，已成功应用于果蔬、畜禽产品、乳制品及饲料等多种食品基质中药物残留的检测，涵盖兽药（如TCs、SAs）、农药（如OPPs、新烟碱类）及部分人用药物。通过TFME、IT-SPME、DSPME、涂层纤维SPME、SBSE等多样化模式与色谱/质谱联用，可有效应对食品基质中蛋白质、脂类等干扰成分的影响，实现痕量残留的高灵敏度分析。其LOD值多低至μg/L级甚至ng/L级，回收率多维持在70%~120%范围内。同时，通过组合表面印迹技术、纳米印迹技术及多功能单体印迹策略、刺激响应印迹策略等优化材料性能（如双功能单体提升极性药物识别效率，热敏材料实现吸附-解吸可控调节），为食品供应链中各种药物残留的高效筛查提供了可靠技术支撑，助力食品安全监管与质量控制。

### 3.2 环境监测

环境监测作为评估环境质量的重要手段，通过系统性测量与分析为环境保护提供科学依据。MI-SPME作为一种先进的样品前处理技术，已广泛应用于水体、土壤等环境基质中药物残留的检测^［52，117-119，121-123］^，显著提升了检测效率与准确性。为环境保护管理、生态风险评估及科学决策制定提供了有力支持，对维护生态环境平衡、实现可持续发展具有重要作用。

Barahona等^［119］^以恩诺沙星（ENRO）为模板分子、MAA为功能单体、EGDMA为交联剂，将MIPs固定在聚丙烯中空纤维（HFs）的孔隙中，成功合成了MIP-HFs复合材料。建立了基于该复合材料的选择性IT-SPME方法，结合HPLC-UV，用于地表水和地下水环境中氟喹诺酮类（FQs）抗生素残留的检测。该方法的回收率为9.4%~24.5%，RSD值小于20%（*n*=3），具有良好的稳定性和准确性。LOD范围为0.1~10 μg/L。该研究为环境监测中FQs等新污染物的检测提供了有效手段。

Monnier等^［117］^使用不同的深共晶溶剂（DES）替代传统的有害有机溶剂（如甲苯），以熔融石英毛细管作为模具，采用虚拟模板印迹策略，以丙嗪为虚拟模板、甲酸和L-薄荷醇（物质的量比1∶1）的混合物为DES致孔剂，发展了一种用于三嗪类化合物的整体式IT-SPME方法。该新型萃取纤维IT-SPME结合HPLC-DAD，用于土壤样品中4种三嗪类化合物的萃取分析，回收率为75.7%~120.1%，LOD为6.2~15.7 ng/g。该研究不仅证实了DES在环境分析中的应用价值，同时推动了绿色印迹技术的发展，为环境监测领域提供了兼具环保性和高效性的新型样品前处理方法。

Chen等^［120］^以GO/MIPs为吸附剂，发展了一种提取海洋环境中诺氟沙星（NOR）的新型样品前处理方法。该GO/MIPs以NOR为模板分子，采用沉淀聚合法与表面印迹技术，在GO表面合成MIPs。将合成的GO/MIPs装入等边三角形中，折叠成聚丙烯膜，并建立MI-SPME包体（MISPME-p），其材料的制备和SPME流程见图3。结合HPLC-DAD测定，加标回收率为90.1%~102.7%，EF为7.01，RSD为2.06%~5.29%。海水和鱼类样品中的LOD分别为0.15 μg/L和0.10 μg/L。该方法具有良好的萃取效率和可重复使用性，成功应用于海水和鱼类样本的检测，为海洋环境中痕量抗生素分析提供了可靠支持。

**图3:**
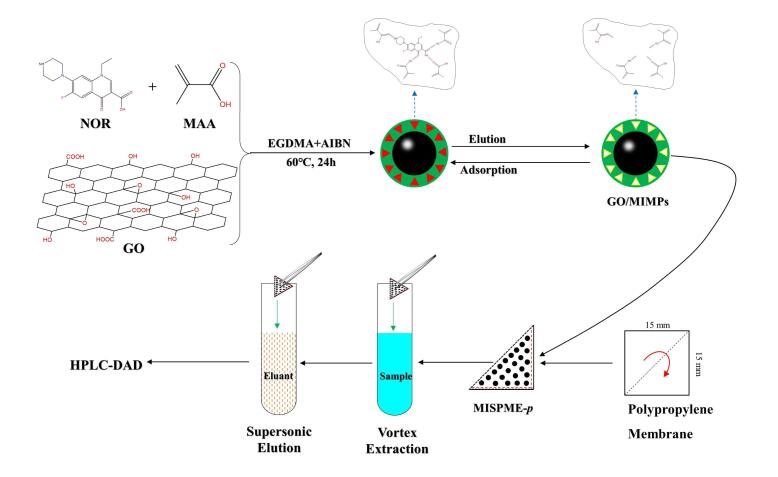
GO/MIPs的制备路线和SPME流程示意图^［120］^

在环境监测领域，MI-SPME技术凭借对复杂环境基质的高效处理能力，成功应用于水体（如地表水、地下水）和土壤中药物残留的检测，涉及FQs、三嗪类化合物等多种污染物。其核心优势在于通过IT-SPME、DSPME等模式与色谱/质谱联用，有效克服环境样品中基质干扰，实现痕量残留的高灵敏测定。其LOD值多低至μg/L级甚至ng/L级，回收率多保持在75%以上，且通过引入DES等绿色溶剂、石墨烯复合MIPs等，兼顾了检测效率与环保性。该技术为环境污染物的快速筛查、生态风险评估提供了可靠的前处理手段，助力环境保护与可持续发展决策。

### 3.3 生物医药

生物医药是指利用生物体、生物组织或其成分进行药物研发与生产的领域，其核心目标在于阐明药物在人体内的代谢机制、精准评估药物疗效，并确保临床用药的安全性与合理性。近年来，MI-SPME技术已广泛应用于尿液、血浆、血清及植物等生物样品^［27，53，63，68，71，73，85，97，124，125，127-131］^，实现了药物的高效特异性检测。

Rahimi等^［124］^采用MIP纤维涂层式SPME结合HPLC-UV技术，建立了一种高选择性检测尿液中痕量大黄酚的分析方法。以大黄酚为模板分子、乙烯基咪唑（Ⅵ）为功能单体、黄蓍胶（TG）为天然交联剂，通过自由基聚合和表面印迹技术，在聚多巴胺功能化不锈钢丝表面构建了耐用、稳定的MI-SPME纤维涂层研究结果表明，该方法的最大吸附量为131.77 ng，LOD为2.68 ng/mL，LOQ为8.95 ng/mL，加标样品的回收率为94.01%~96.20%，纤维内和纤维间的RSD分别为4.30%和5.06%。在0.007 1~0.2 μg/mL范围内呈现良好线性关系，并成功用于尿液中大黄酚的萃取和测定。上述结果验证了该方法准确、高效、简便且经济的特点，在生物样品药物检测领域展现出巨大潜力，为相关研究和临床实践提供了技术支持，有望进一步推动生物医药领域的发展。

Ansari等^［125］^以OXY为模板分子、MAA为功能单体，采用表面印迹技术制备了基于磁性GO和碳点纳米粒子的MIPs（MIP@MGO/CDs NPs）。该MIPs结合超声辅助DSPME（UA-DSPME）与HPLC-UV，实现了人体尿液中OXY的选择性萃取。结果表明，该MIPs的最大吸附量达99 mg/g，印迹因子为3.39。在优化条件下，LOD为0.80 ng/mL，LOQ为2.67 ng/mL，线性范围为1~2 000 ng/mL；人尿中OXY的回收率为92.50%~103.20%，RSD<3.65%。该方法具有快速、低成本、宽线性范围和高灵敏等特点，为复杂生物样品中OXY的识别和分离提供了新颖、高效且便捷的技术手段，降低其在临床应用中的局限性，提高用药安全性。

Tu等^［53］^采用RAFT沉淀聚合（RAFTPP）技术，以MAA为功能单体、EGDMA为交联剂，合成了具有密集接枝聚甲基丙烯酸羟乙酯（PHEMA）刷的新型MIPs微球。通过在引发剂AIBN存在下将PHEMA大分子链转移剂（macro-CTA）与MIPs微球偶联，制备了亲水性MIPs材料。将其用作DSPME吸附剂，结合HPLC-UV，实现了从未稀释的复杂牛血清样品中直接、选择性捕获普萘洛尔。该方法回收率为85.2%~97.4%，准确度较高（RSD为2.3%~3.7%）；LOD为0.002 μmol/L，LOQ为0.006 7 μmol/L，在0.01~100 μmol/L范围内的线性相关系数为0.999 4。日内和日间分析均证实了方法的高精度，该亲水MIPs的可重用性良好。该研究表明基于MIPs的亲水性DSPME吸附剂在快速、准确、可靠测定复杂生物样品中药物含量方面的巨大潜力，有望促进对普萘洛尔等药物在体内代谢过程的深入理解和为临床用药提供科学依据。

在生物医药领域，MI-SPME技术针对尿液、血浆、血清等复杂生物基质，实现了大黄酚、羟考酮、普萘洛尔等药物残留的高选择性检测。凭借MIPs材料的特异性识别能力，结合涂层纤维SPME、超声辅助DSPME等模式，有效消除生物基质中蛋白质、脂质等成分的干扰，与色谱/质谱联用，LOD可达ng/L级，回收率普遍在85%以上。同时，亲水性MIPs、刺激响应型MIPs等的应用，进一步提升了对生物样品中极性药物、微量成分的捕获效率，为药物代谢研究、临床用药安全监测及毒理学分析提供了高效、精准的技术支撑，助力生物医药领域的精准检测与科学决策。

## 4 总结与展望

MI-SPME技术在食品、环境和生物样品等多种复杂介质的样品前处理中获得了显著效果，结合色谱/质谱检测，在药物残留分析中展现出巨大应用潜力，为食品安全、环境监测、生物医药等领域相关研究提供了可靠支撑。同时，MI-SPME技术仍面临诸多挑战和发展机遇，可从以下方面进行优化与创新。

在MIPs的制备与优化方面，尽管基于MIPs的吸附剂在实用性和适应性方面表现良好，但仍面临模板分子泄漏、识别位点分布不均及环境稳定性差等挑战^［42］^。为克服这些限制，需优化制备工艺。一方面，可利用密度泛函理论（DFT）或分子动力学模拟筛选功能单体与交联剂的最佳配比^［132-134］^，以优化MIPs的三维结构和吸附性能；另一方面，通过引入新型印迹策略，如虚拟模板、多模板印迹、刺激响应（如pH、温度、光、磁）^［24］^及电化学印迹技术^［135］^，可进一步提升MIPs的选择性和环境适应性。此外，采用点击化学交联^［80］^、光引发聚合^［136］^等可控交联方法，可增强MIPs的机械强度和结构稳定性。

在MI-SPME模式创新方面，传统纤维涂层式和整体柱式SPME虽具有高选择性和稳定性，但仍受限于传质效率低、制备复杂及成本高等问题，尤其在复杂基质中识别力有限^［75，137］^。为此，研究人员正致力于开发新型涂层材料和萃取头结构，例如，基于纳米粒子的MIPs涂层（如磁性纳米粒子）可提高萃取效率并简化磁分离操作^［71］^；石墨烯及其衍生物（如GO等）凭借高比表面积和优异稳定性^［125］^，可显著增强MIPs的识别与萃取能力^［138］^；介孔分子筛（如MCM-41、SBA-15）因其高度有序的孔道结构、可调的孔径和丰富的表面修饰位点，为MIPs提供了理想载体^［139］^。通过将MIPs与介孔分子筛复合，可有效解决传统印迹材料传质阻力大、位点包埋深的问题，同时提升对大分子药物（如抗生素、蛋白质类残留）的吸附容量和动力学性能。此外，MOFs^［115］^、共价有机骨架（COFs）^［80］^和氢键有机骨架（HOFs）^［140］^等多孔骨架材料不仅具有高度的结构可调性和化学稳定性，还能通过精确控制孔径和表面化学性质实现对目标分子的高效捕获和分离，从而极大扩展MI-SPME技术的应用范围。然而，新型材料应用亦存在挑战。如，纳米团聚影响效率、石墨烯制备成本高且工艺复杂；介孔分子筛的合成虽相对成熟，但其在MI-SPME中的功能化修饰（如氨基化、巯基化）仍需优化反应条件以避免孔道堵塞；多孔骨架材料的合成条件苛刻，对实验设备和工艺要求高，目前还缺乏成熟的大规模生产工艺。未来研究需优化制备工艺、降低成本，并探索高效集成策略。

此外，MI-SPME与自动化技术的融合是重要发展趋势。通过集成微流控技术、智能控制系统和便携式检测设备，可构建高效自动化的检测平台，不仅提升药物残留分析的准确性和效率，还可实现现场快速检测，推动食品安全、环境监测和生物医药等领域的革新。同时，作为SPME技术核心联用平台的色谱/质谱检测技术本身也在快速发展。HPLC-高分辨率质谱法（HPLC-HRMS）^［141］^、气相色谱-高分辨率质谱法（GC-HRMS）^［142］^以及气相色谱-离子迁移谱（GC-IMS）^［143］^等新兴联用技术将持续提升检测的灵敏度、选择性以及对复杂化合物（如异构体）的解析能力。色谱/质谱仪器的小型化与便携化（如Mini-MS， Portable GC-MS/LC-MS）^［144，145］^以及微流控芯片-质谱联用技术^［146］^的进步，为现场快速检测提供了强大的核心工具。未来，MI-SPME技术有望与高性能便携式/小型化色谱/质谱仪结合，实现即时检验（POCT），为临床诊断和体内监测提供即时、准确的数据支持，大大提高诊断效率和准确性。同时，绿色可持续发展理念也需贯穿MI-SPME技术的研发与应用。该技术本身具有溶剂用量少的优势，符合绿色化学原则，但未来仍需优化MIPs涂层的环保制备工艺，如通过使用DES等绿色溶剂^［103］^或生物可降解材料，并开发高效的回收再利用技术等。

综上所述，MI-SPME技术在药物残留检测中具有广阔的应用前景。未来，随着制备工艺的优化、新型材料的开发、自动化技术的融合，以及POCT和绿色化学理念的深入实施，该技术在食品安全、环境监测、生物医药及商业化应用等领域发挥更大作用。
